# Chronic and infectious respiratory mortality and short-term exposures to four types of pollen taxa in older adults in Michigan, 2006-2017

**DOI:** 10.1186/s12889-025-21386-3

**Published:** 2025-01-16

**Authors:** Peter S. Larson, Allison L. Steiner, Marie S. O’Neill, Alan P. Baptist, Carina J. Gronlund

**Affiliations:** 1 Social Environment and Health Program, Institute for Social Research, University of Michigan, 426 Thompson St., Ann Arbor, MI 48104 USA; 2 Department of Epidemiology, School of Public Health, University of Michigan, 123 Observatory, Ann Arbor, MI 48104 USA; 3 Climate and Space Sciences and Engineering, University of Michigan, 2455 Hayward St., Ann Arbor, MI 48109 USA; 4 Department of Environmental Health Sciences, School of Public Health, University of Michigan, 123 Observatory, Ann Arbor, MI 48104 USA; 5 Division of Allergy and Clinical Immunology, Henry Ford Health, 1 Ford Place, Detroit, MI 48202 USA; 6 Health Behavior and Health Education, University of Michigan School of Public Health, University of Michigan, 123 Observatory, Ann Arbor, MI 48104 USA

**Keywords:** Respiratory mortality, Pollen, Environmental health, Respiratory health

## Abstract

**Introduction:**

Levels of plant-based aeroallergens are rising as growing seasons lengthen and intensify with anthropogenic climate change. Increased exposure to pollens could increase risk for mortality from respiratory causes, particularly among older adults. We determined short-term, lag associations of four species classes of pollen (ragweed, deciduous trees, grass pollen and evergreen trees) with respiratory mortality (all cause, chronic and infectious related) in Michigan, USA.

**Methods:**

We obtained records for all Michigan deaths from 2006-2017 from the Michigan Department of Health and Human Services (MDHHS). Deaths from infectious and chronic respiratory-related causes were selected using International Classification of Diseases (ICD-10) codes. Pollen data were obtained from a prognostic model of daily pollen concentrations at 25 km resolution. Case-crossover models with distributed lag non-linear crossbases for pollen were used to estimate associations between lags of daily pollen concentrations with mortality and to explore effect modification by sex and racial groups.

**Results:**

127,163 deaths were included in the study. Cumulative daily high concentrations (90th percentile) of deciduous broadleaf, grass and ragweed were associated with all-cause respiratory mortality at early lags with e.g., a 1.81 times higher risk of all respiratory deaths at cumulative 7 day lag exposure to deciduous broadleaf pollen at the 90th percentile (95% confidence interval: 1.04, 3.15). Exposure to high concentrations of grass and ragweed pollens was associated with increased risk for death from chronic respiratory causes. No association was found for any pollen species with death from infectious respiratory causes though there was a positive but non-significant association of exposure to deciduous broadleaf and ragweed pollens. We found no evidence to suggest effect modification by race or sex.

**Conclusions:**

Modelled exposures to high concentrations of pollen taxa were associated with increased all-cause and chronic respiratory mortality among older adults. Results suggest that pollen exposure may become more important to respiratory mortality as the temperatures increase and pollen seasons lengthen.

**Supplementary Information:**

The online version contains supplementary material available at 10.1186/s12889-025-21386-3.

## Introduction

Anthropogenic climate change is altering temperature and precipitation patterns, resulting in profound changes to regular pollen seasons in North America [1, 2] and elsewhere [3–5]. Consequently, people now not only experience longer and more intense exposures to aeroallergens [1, 6] but greater numbers of people are being exposed due to rapid increases in the geographic range of various plant species [7]. Both might have important present and future implications for public health. However, the effects of exposure to pollens might vary by pollen taxa, and cumulative effects of exposure over the short term to specific pollens that could be associated with specific health risks.

Exposure to aeroallergens is associated with a number of respiratory-related health risks [8] which include both chronic and infectious health conditions. One such health condition is allergic rhinitis, a heterogeneous disorder that includes hypersensitivity to a number of allergens including pollens leading to acute and long term physical reactions [9]. Allergic rhinitis has been associated with coronary heart disease and myocardial infarction, possibly due to heightened inflammation [10], though results have been inconsistent [11]. However, allergic rhinitis is a major risk factor for asthma and asthma related exacerbations [12–14] and risk for mortality from asthma is highest among older adults with asthma compared to those of younger ages [15]. For infectious respiratory mortality, the evidence is mixed. Some research has suggested protective effects of pollen exposure on the incidence of flu-like illnesses [16, 17]. However, a multi-country study demonstrated that risk for SARS-CoV-2 infection was positively correlated with increased exposure to pollens [18]. To date, however, little research exists on how short term lag exposure to specific pollen classes might impact patterns of mortality from infectious and chronic respiratory causes.

Changes in yearly pollen exposure patterns might result in protracted impacts on the public health. Lengthened pollen seasons and increased pollen loads for multiple pollen taxa [19] could result in increased duration and magnitude of health risks. As an example, research has indicated that emergency department visits for chronic respiratory conditions such as asthma and allergic asthma related concerns will increase with increasingly intense oak pollen seasons [20, 21]. Formal tests of associations of short term lag exposures to multiple types of pollens with specific health outcomes with demonstrated links to allergic rhinitis would contribute to knowledge of how climate change might impact the public health. This research seeks to fill that gap.

With this research, we hypothesize that short term, lag exposures to plant-based aeroallergens will be associated with increased risks for mortality from respiratory causes. We expect that patterns of association will vary by pollen class and by type of respiratory cause. We expect that patterns of association between specific pollen types will differ between chronic and infectious related respiratory mortality. We also expect there to be variation in the direction and/or magnitude of association of pollen exposure and mortality (all, chronic and infectious) between men and women and Black/white racial groups. We conduct this analysis using official death records from the State of Michigan and a novel prognostic model based raster data set of four pollen taxa in North America. We consider our sample to be an ideal population to conduct this research as 1) Michigan is broadly representative of many other Midwestern US States and other industrialized areas of the world and 2) Michigan’s flora is such that Michigan residents are exposed to many of the most important allergenic pollen species.

## Materials and methods

### Mortality data

This research used mortality records for Michigan residents obtained from by the Michigan Department of Health and Human Services (MDHHS) for the years 2006–2017. The MDHHS records and maintains information for all deaths which occur within the borders of the State of Michigan. These records include information such as age, sex, residential address, date, cause and whether the death occurred at a hospital. For hospital based deaths, there was no information on how long the decedent had been hospitalized prior to death. We included decedents if the primary cause of death was due to respiratory causes per the ICD-10 code listed on the death record (J00-J99). We categorized the causes of death as chronic lower respiratory causes (e.g. asthma) (J40-J47) and death from acute upper respiratory infections (J00-J06), influenza and pneumonia (J10-J18) and other acute lower respiratory infections (J20-J22). We further subsetted the dataset into specific conditions of interest: deaths from chronic obstructive pulmonary disease (J44) and deaths from influenza (J09-J11). No information on prior medical treatment or pharmaceutical use is included in MDHHS death records.

### Environmental data

Data on estimated atmospheric concentrations of speciated plant-based aeroallergens were produced using a prognostic pollen emissions model for 2006–2017 [22]. The pollen emissions model provides daily count estimates of four pollen taxa: deciduous trees (including *Acer* (maple), *Alnus* (alder), *Betula* (birch), *Fraxinus* (ash), *Morus* (mulberry), *Platanus* (sycamore), *Populus* (poplar), *Quercus* (oak), and *Ulmus* (elm)), evergreen trees (including the *Cupressaceae* and *Pinaceae* families), grasses, and ragweed. Data are raster layers that account for geography, vegetation, and meteorological parameters. The model covers the continental United States at a 25-km grid resolution [22]. A goal of this research was to use spatio-temporal model based estimates of pollen exposure as a suggested method to improve common, monitor based of pollen exposure measurements. Problem with data from pollen monitors such as those managed by the National Allergy Bureau (NAB) include long distances from the actual location of exposure to the nearest pollen monitor, which can be hundreds of miles away and incomplete and missing data on a temporal scale [23]. Though model based data is not without limitations, the data used for this research allows for temporally complete estimates at a finer spatial scale. We wish to demonstrate that these data might be a promising new method of testing associations of pollen exposure and health outcomes.

### Statistical methods

Locations of exposures were based on the decedent’s listed residential address. Address were geocoded to latitude and longitude coordinates. Pollen exposures for all four pollen taxa were extracted to the mortality database based on latitude-longitude locations. To allow for testing of lag exposure, daily concentrations for all four pollen taxa up to 14 days preceding the date of death were extracted for each individual at the location of residence. Decedents with residences outside the State of Michigan were excluded from the analysis.

We used a time-stratified case crossover design to assess associations between each pollen species and each classification of respiratory mortality (all, chronic, and infectious), in separate models. The case crossover design is effective for evaluation of acute-onset outcomes in response to short-term exposures [24–26]. In this design, each individual serves as their own control, with case days, i.e., the date of death, and control days. Control-days are set to be the same day-of-the-week within the same month and year as the case date of death following approaches used in other studies [27, 28] and assigned environmental exposures and lags accordingly. The method allows us to account for seasonality and possible temporal autocorrelation by matching case and control days in small time windows [29]. The case crossover design also controls for all non-time-varying characteristics such as sex or race by design [24].

Associations of environmental exposures with respiratory mortality were estimated using the distributed lag non-linear model (DLNM) regression framework. DLNMs offer a means of assessing risk as a function of both timing and intensity of exposure. DLNMs are based on the definition of a cross-basis, which is comprised of two functions which describe exposure-responses and the lag structure [30]. Using the cross-basis in a defined statistical model structure, associations between exposures and outcomes are estimated and then parameter estimates and standard errors are used to predict risk across exposure levels and lag days.

Descriptive measures of time-invariant variables and temporal patterns of all respiratory mortality and all environmental measures were explored as a first step in the analysis. Possible non-linearity was graphically examined for all time-variant environmental measures. Based on these findings, natural cubic spline parameterization using three degrees of freedom for each exposure and two degrees of freedom for the lags was deemed appropriate.

As the time between exposure and death likely varies due to comorbidities and the nature and type of health care provided, we explored the effects of pollen aeroallergens over a 14-day period, reporting associations with the same day’s exposure and lag days 1, 7, and 14. As pathologies of exposure to aeroallergens and allergic rhinitis (AR) differ between men and women [31], and might also differ by race as a result of pre-existing susceptibility or geography, we explored possible effect modification by sex and race.

## Results

There were 127,163 deaths from respiratory causes between January 1, 2006 and December 31, 2017. Of these, 65,412 (51.4%) were female. Whites comprised nearly 90% of deaths in Michigan, followed by Blacks (9.42%). American Indians and Alaskan Natives, Asians and other groups comprised less than 2% of deaths. Due to the small numbers of deaths in these other groups, we excluded non-Blacks and non-Whites from this analysis. Deaths from chronic lower respiratory and infectious respiratory causes comprised 61.2% and 19.9% of respiratory deaths, respectively. COPD was associated with 54.9% of respiratory deaths. Influenza was noted as a cause for only 1.39% of all respiratory deaths. Given the small number of deaths associated with influenza, we opted to exclude them from further analysis. See Table 1.

**Table 1:** Summary descriptive statistics of the 127,163 deaths from respiratory related causes in the State of Michigan between 2006 and 2017

	Group N (%)	
Sex:		
Male	61,751 (48.6%)	
Female	65,412 (51.4%)	
Age (mean (sd))	76.5 (14.1)	
Race or ethnic group:		
White	113,096 (88.9%)	
Black or African American	11,976 (9.42%)	
American Indian or Alaskan Native	790 (0.62%)	
Asian	167 (0.13%)	
Other	1134 (0.89%)	
All chronic lower respiratory:	77820 (61.2%)	127,163
COPD:	69858 (54.9%)	
Asthma:	1507 (1.19%)	
All infectious respiratory:	25245 (19.9%)	
Influenza:	1769 (1.39%)	

Spatial and temporal patterns of exposure in Michigan vary by pollen class (see Supplemental Figures 1 and 2). Deciduous broadleaf and evergreens release pollen in the Spring. Grass pollens are most prevalent in late Spring to early Summer. Ragweed pollen concentrations are highest in the Fall. To rule out possible additive or multiplicative effects of exposure to multiple taxa at once we calculated Pearson correlations of daily pollen concentrations of all four taxa. We found that temporal patterns of taxa concentrations were weakly correlated with one another (results not shown).

### Associations of speciated pollens with respiratory related mortality

Associations between all four classes of speciated pollens and respiratory mortality (all cause, chronic and infectious) were estimated. We initially attempted to include precipitation, temperature and air pollution (PM 2.5) in the models, suspecting a possible confounding relationship between both mortality and the exposure. However, the inclusion of these variables led to unstable parameter estimates due to multicollinearity, as precipitation and temperature are both used to determine the modeled pollen concentrations. PM2.5 was correlated with both temperature and precipitation as has been shown in other research [32] and thus also correlated with pollen to varying extents (results not shown).

We present odds ratios for the odds of death at high levels of cumulative exposure (90th percentile) vs. no exposure for lag days 0, 0–1, 0–7 and 0–14. Deciduous broadleaf pollen exposure was associated with increased odds of mortality at lags 0–1 (cumulative OR 1.14 95% CI (1.02, 1.27)) and lags 0–7 days (cumulative OR 1.81 95% CI (1.04, 3.15)). Exposure to evergreen pollens was not significantly associated with respiratory mortality at any lag. Grass pollens were weakly associated with all-cause and chronic respiratory mortality at early lags. A significant protective effect of grass pollens on all respiratory (cumulative OR 0.55 95% CI (0.41, 0.73)) and of infectious respiratory mortality (cumulative OR 0.52 95% CI (0.28, 0.98)) was found at two weeks . Exposure to ragweed up to lag 1 was significantly associated with all respiratory mortality (cumulative OR 1.31 95% CI (1.11, 1.56)), and for chronic respiratory mortality up to one week (cumulative OR 2.16 95% CI (1.22, 3.80)). Further, odds of death from COPD was associated with ragweed exposure up to one week (cumulative OR 2.07 95% CI (1.14, 3.77)). See Table 2 for full results.

**Table 2:** Odds ratios and 95% confidence intervals of cumulative associations of exposure to the 90th percentile (compared to no exposure) of modelled pollen concentrations with all cause respiratory mortality, mortality from causes associated with chronic respiratory illnesses, COPD and infectious respiratory causes

Exposure	Lag	All	Chronic	COPD	Infectious
Deciduous broadleaf					
	0	**1.14 (1.02, 1.27)**	1.11 (0.97, 1.28)	1.12 (0.97, 1.29)	1.05 (0.84, 1.32)
	1	**1.27 (1.04, 1.56)**	1.22 (0.95, 1.58)	1.23 (0.94, 1.62)	1.1 (0.72, 1.69)
	7	**1.81 (1.04, 3.15)**	1.61 (0.81, 3.22)	1.61 (0.78, 3.34)	1.32 (0.42, 4.11)
	14	1.35 (0.63, 2.91)	1.18 (0.45, 3.12)	1.1 (0.4, 3.04)	1.31 (0.27, 6.34)
Evergreen					
	0	0.9 (0.64, 1.27)	0.92 (0.62, 1.37)	0.9 (0.57, 1.41)	0.79 (0.32, 1.98)
	1	0.83 (0.44, 1.58)	0.86 (0.41, 1.82)	0.83 (0.36, 1.96)	0.63 (0.11, 3.52)
	7	0.65 (0.11, 3.97)	0.84 (0.1, 7.07)	0.98 (0.09, 11.16)	0.16 (0, 15.94)
	14	0.88 (0.06, 13.4)	1.86 (0.07, 46.9)	4.45 (0.12, 162.75)	0.04 (0, 67.12)
Grass					
	0	**1.03 (1, 1.07)**	**1.05 (1, 1.1)**	1.04 (0.99, 1.09)	1.02 (0.94, 1.1)
	1	1.06 (0.98, 1.13)	**1.09 (1, 1.19)**	1.07 (0.97, 1.18)	1.02 (0.88, 1.19)
	7	0.97 (0.8, 1.18)	1.1 (0.85, 1.42)	1.06 (0.81, 1.39)	0.89 (0.58, 1.38)
	14	**0.55 (0.41, 0.73)**	0.69 (0.47, 1)	0.69 (0.46, 1.02)	**0.52 (0.28, 0.98)**
Ragweed					
	0	**1.16 (1.06, 1.27)**	**1.25 (1.11, 1.4)**	**1.25 (1.11, 1.41)**	1.09 (0.93, 1.28)
	1	**1.31 (1.11, 1.56)**	**1.5 (1.21, 1.86)**	**1.5 (1.2, 1.88)**	1.17 (0.86, 1.59)
	7	1.51 (0.96, 2.38)	**2.16 (1.22, 3.8)**	** 2.07 (1.14, 3.77)**	1.55 (0.69, 3.48)
	14	0.5 (0.26, 0.93)	0.65 (0.3, 1.43)	0.56 (0.24, 1.28)	1.43 (0.45, 4.54)

Bolded entries are statistically significant at p <.05

**Fig. 1:**
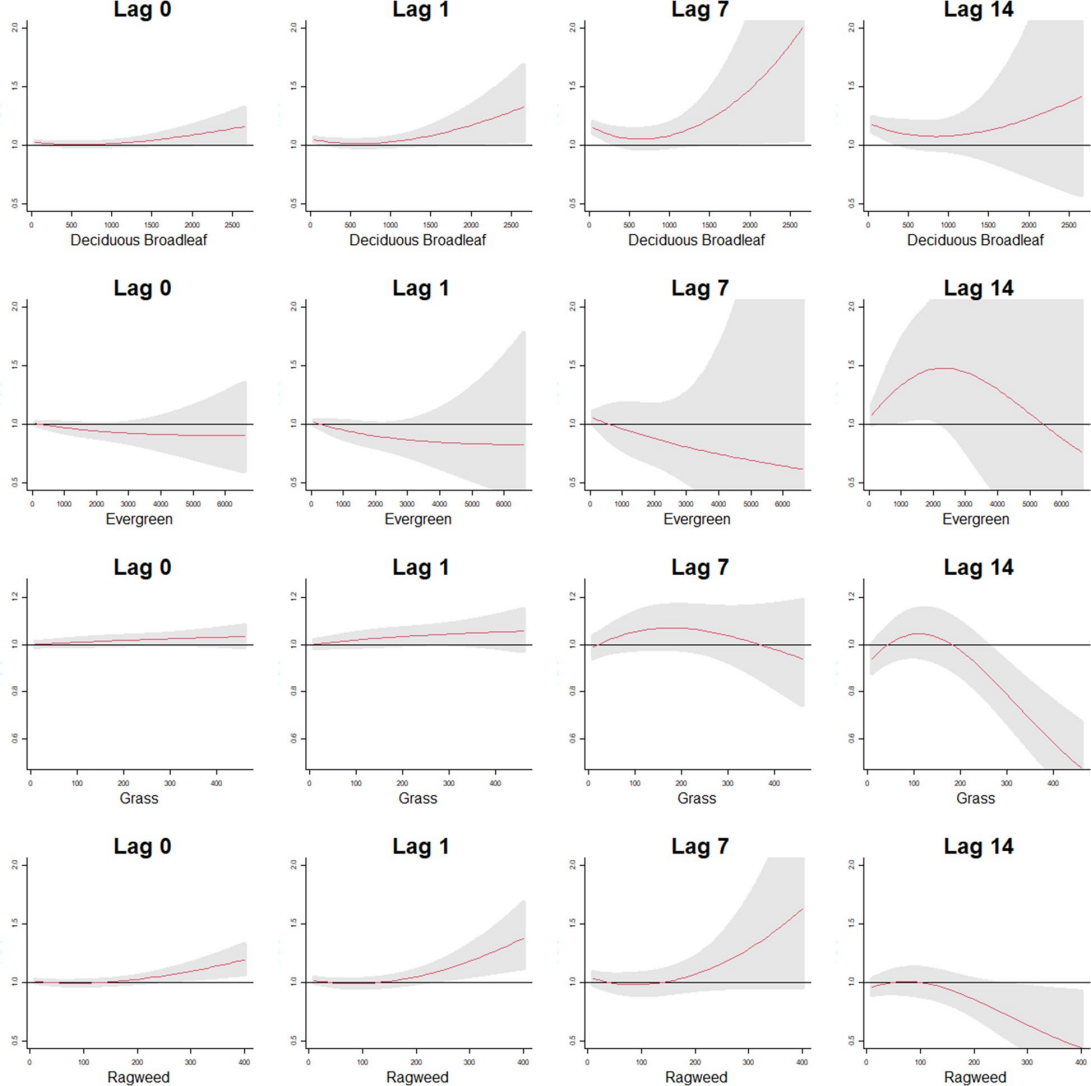
Cumulative exposure-response patterns for the four pollen taxa at the 0, 1, 7 and 14 lag day exposures for all respiratory related deaths

**Fig. 2:**
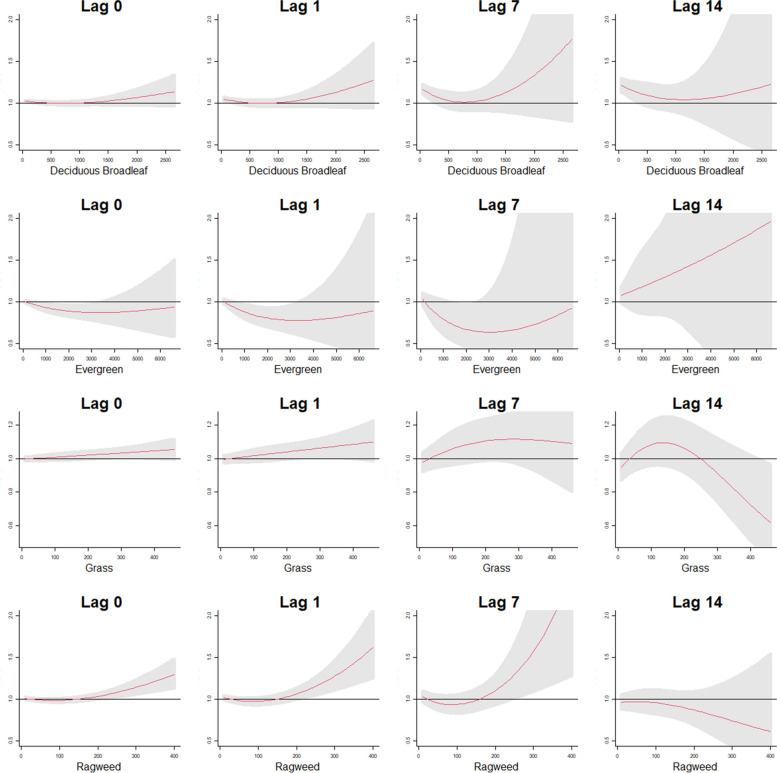
Cumulative exposure-response patterns for the four pollen taxa at the 0, 1, 7 and 14 lag day exposures for deaths associated with chronic respiratory causes

**Fig. 3:**
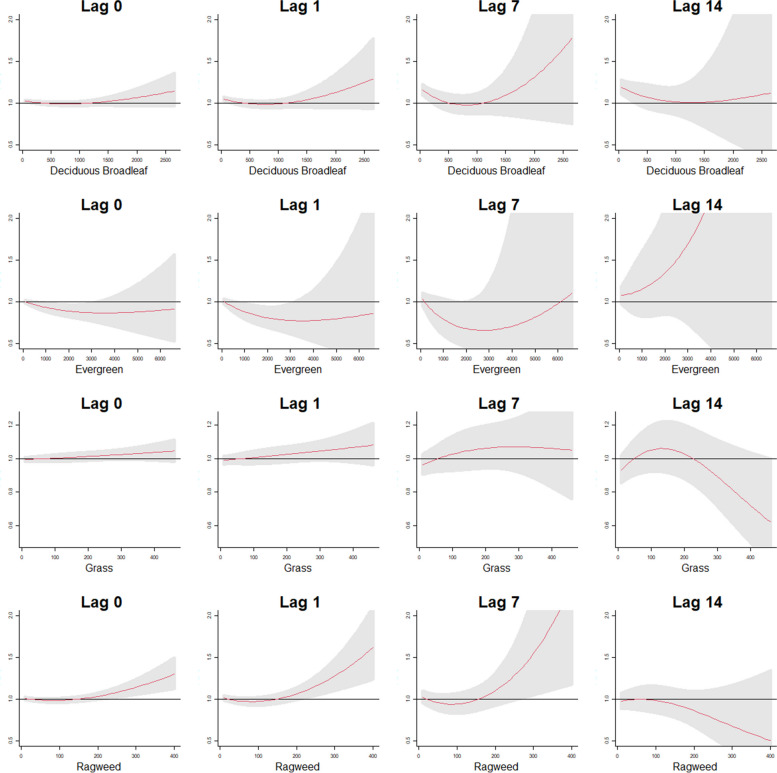
Cumulative exposure-response patterns for the four pollen taxa at the 0, 1, 7 and 14 lag day exposures for deaths associated with COPD

**Fig. 4:**
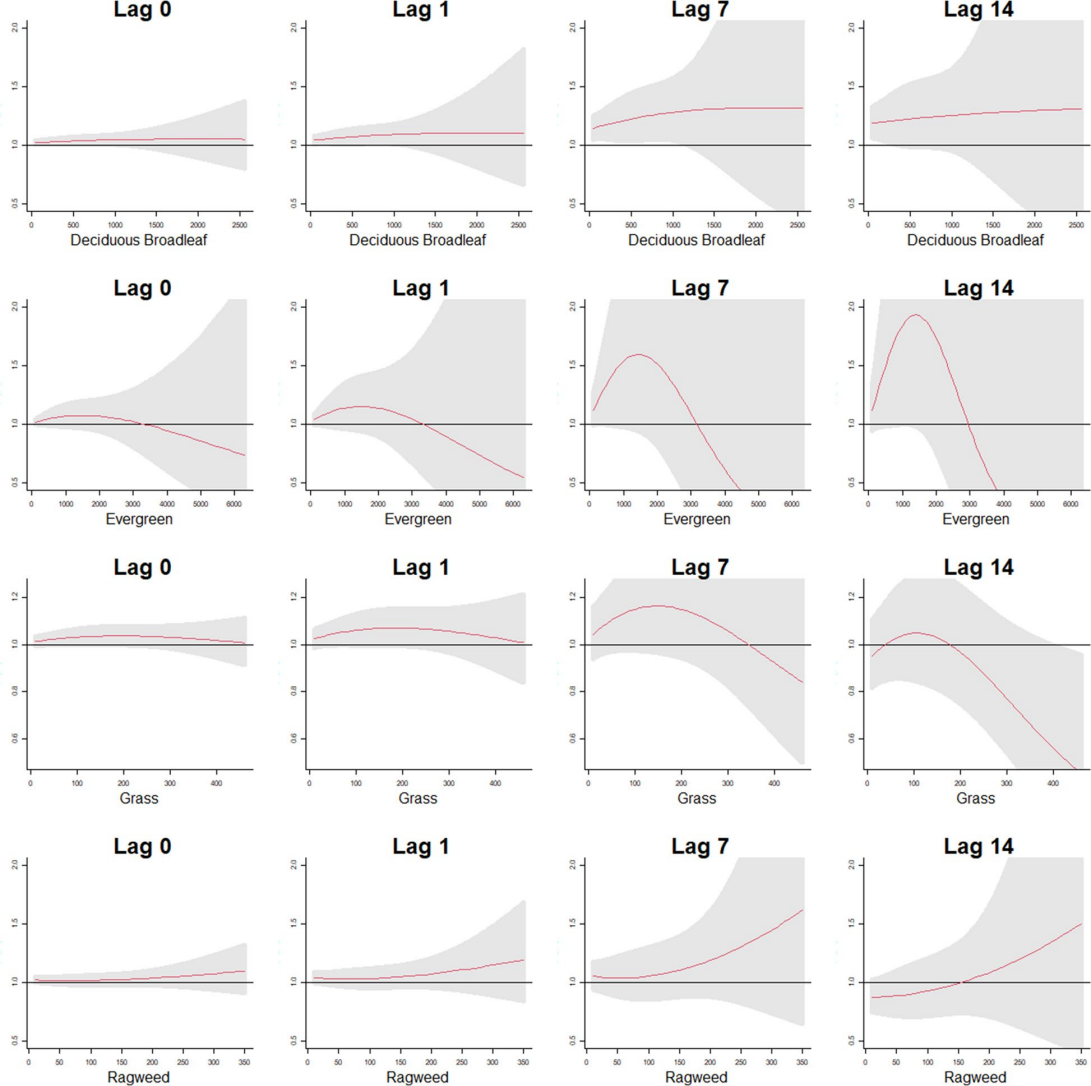
Cumulative exposure-response patterns for the four pollen taxa at the 0, 1, 7 and 14 lag day exposures for deaths associated with infectious respiratory causes

Figure 3 shows exposure-lag-response patterns for all four pollen species. Mortality risk increased for high concentrations of deciduous broadleaf and evergreen pollen concentrations over time. Figures 1, 2, 3 and 4 show exposure-response patterns for the four pollen taxa at same day and cumulative 1, 7 and 14 days of exposure for all cause respiratory, mortality from chronic respiratory causes, mortality from COPD related causes and from infectious respiratory causes, respectively. The odds of death from respiratory causes increased with increasing exposure to ragweed pollen at high levels of exposure. Grass pollens showed a protective effect at high levels of exposure at lag day 14. For chronic respiratory and COPD related deaths, exposure to high levels of ragweed was associated with elevated odds of mortality. While overall, there was no evidence to suggest a statistically significant association of pollen exposure with deaths from infectious respiratory causes, there was a positive though non-significant association of ragweed exposure with mortality.

### Effect modification by listed sex and race

We tested for effect modification of the pollen/mortality association by sex and Black/white race, except for evergreen pollen and race, for which we had an inadequate number of individuals of Black race in regions with evergreen pollen exposure. There was no evidence to suggest effect modification by race or sex, though we found protective associations of grass with all four mortality groups for women and for white people for all types of respiratory deaths. Associations of grass pollen taxa with chronic and infectious respiratory mortality differed in direction by sex groups. See Table 3 for full results.

**Table 3:** Odds ratios of selected lag associations of exposure to the 90th percentile (compared to no exposure) of speciated pollen concentrations with respiratory mortality by gender and Black/white race

	Outcome	Group	Db	Eg	Gra	Rag
1	All	Male	1.47 (0.54, 4.01)	4.26 (0.1, 174.87)	0.79 (0.52, 1.2)	0.47 (0.22, 0.99)
2		Female	1.23 (0.42, 3.59)	0.09 (0, 6.23)	0.4 (0.27, 0.59)	0.62 (0.26, 1.49)
3		White	1.5 (0.68, 3.32)		0.54 (0.4, 0.73)	0.58 (0.3, 1.12)
4		Black	0.11 (0.01, 1.85)		0.64 (0.25, 1.59)	0.31 (0.07, 1.44)
5	Chronic	Male	1.53 (0.48, 4.86)	3.93 (0.06, 251.2)	1.06 (0.61, 1.84)	0.54 (0.22, 1.31)
6		Female	0.88 (0.23, 3.34)	0.41 (0.01, 27.67)	0.47 (0.28, 0.79)	0.92 (0.31, 2.73)
7		White	1.28 (0.47, 3.46)		0.69 (0.47, 1.02)	0.78 (0.35, 1.77)
8		Black	0.42 (0.15, 1.21)		0.65 (0.17, 2.51)	0.27 (0.03, 2.32)
9	COPD	Male	1.37 (0.45, 4.15)	4.18 (0.05, 366.81)	0.94 (0.53, 1.68)	0.57 (0.22, 1.45)
10		Female	0.85 (0.21, 3.41)	6.09 (0.09, 419.94)	0.52 (0.3, 0.9)	0.65 (0.21, 2.05)
11		White	1.19 (0.42, 3.38)		0.67 (0.44, 1.02)	0.62 (0.26, 1.47)
12		Black	0.45 (0.14, 1.46)		0.96 (0.22, 4.13)	0.33 (0.03, 3.71)
13	Infectious	Male	2.81 (0.3, 26.41)	0.34 (0, 3927.58)	1.38 (0.55, 3.45)	1.48 (0.28, 7.71)
14		Female	0.65 (0.09, 4.89)	0.01 (0, 150)	0.22 (0.09, 0.53)	1.27 (0.31, 5.23)
15		White	1.48 (0.28, 7.7)		0.59 (0.29, 1.17)	1.49 (0.43, 5.1)
16		Black	0.63 (0.16, 2.49)		0.38 (0.07, 1.91)	0.97 (0.05, 18.47)

## Discussion

We found mixed evidence to suggest lag associations between exposure to common classes of pollen species and respiratory related mortality in a temperate, Midwestern state of the United States. Risks varied by exposure level and lag exposure days, suggesting that the timing between exposure and death can be as long as two weeks for extreme levels of deciduous broadleaf and as little as one week for ragweed pollens. Our results agree with other studies suggesting that pollens are associated with respiratory related mortality [33]. Similar to our results, one study in the Netherlands found that *Betula* pollen (also from a deciduous tree) was associated with cardiovascular and respiratory mortality [33]. High concentrations of alder tree pollen, another deciduous species, was also shown in other studies to be associated with an increased odds of death from respiratory causes [34]. We did not find an association of any kind of mortality with evergreen pollen. The associations of the pollen classes with respiratory mortality might vary due to the components included in the prognostic models. For example, in the ragweed pollen class, weeds other than *Ambrosia* were not included in the emissions model due to the lack of land cover information for other types of weed species. However, cumulative exposure to ragweed up to one week was associated with all chronic and COPD associated deaths suggesting that ragweed might be an important focus of research for studies on pollen exposure and those with chronic respiratory health problems.

The mechanisms of how pollens might impact respiratory mortality are not well understood. One possible mechanism that has been suggested is that pollen influence symptomatic outcomes of respiratory infections. For example, one study indicated that pollen might play a role in the seasonality of influenza transmission [16]. Some research has also suggested that pollen exposures weaken innate defenses against respiratory infections [35] but other research suggest possible protective effects against infections such as influenza [17]. Additionally, air pollution may exacerbate the allergenic potential of pollens, potentially worsening symptoms for some and increasing the risk of worse outcomes from respiratory infections [36]. Pollens stimulate the productions of IgE antibodies, which are involved in short and long term allergic inflammation [37]. An increased inflammatory response might increase susceptibility to respiratory infections in sensitive individuals. More research is needed to assess the role that pollen and specific pollen species might play in determining risk for respiratory infections and subsequent outcomes.

We did not find racial differences in associations of pollens with mortality as observed in other studies [38, 39]. Vulnerability to a wide range of allergens is known to be higher in Black individuals than in Whites [40], and multiple factors likely explain this racial disparity. Blacks were found to be more vulnerable to tree, grass and ragweed pollens than other racial groups in a study from Connecticut [41]. Black populations in Michigan, are concentrated in the heavily urbanized areas in and around Detroit, MI. Competing risks from other exposures, e.g., air pollution and molds, may mask associations of mortality with pollen. More work should be done to assess potential competing risks and mediators of racial disparities in risk for respiratory death from exposure to pollen using other types of metrics, including hospital admissions or pharmaceutical prescription records.

We found no evidence to suggest effect modification by sex or race for any type of mortality for exposure to any type of pollen. Research has indicated sex differences in asthma and allergic rhinitis and age-related prevalence shifts between sexes, but little evidence exists supporting that sex differences persist in adulthood [42, 43]. Sex differences have been noted for infectious respiratory mortality for COVID-19 [44], influenza [45] and pneumonia [46]. The significant results for grass exposure could reflect differences in exposure to grass pollens through outdoor occupations and activities such as lawn maintenance or recreation. More work should be done to better assess possible gender disparities in respiratory diseases through gender specific work and recreational activities.

This research has several limitations. While the database records the location of residence, it does not confirm when and for how long the decedent was last present at that location. Older adults might relocate to live with relatives or to care facilities before death. Thus, the pollen exposure estimates likely had some misclassification by space or time. Information on the presence of pre-existing conditions such as asthma or allergic sensitization and use of and adherence to medication treatments (such as anti-histamines) was unavailable in the death records. Future studies might explore associations of pollen on specific health outcomes that might lead to mortality using data on hospital admissions or clinic visits that would enable more careful assessment of the role of pollen in these outcomes and the role of individual factors such as medication use and pre-existing conditions

Another possible limitation of this analysis was related to the model-based estimates of pollen concentrations. The pollen modeling is based on limited pollen count observations (e.g., Michigan had only one site which, at this time, is no longer providing pollen concentrations), and is also limited by the land cover data that represents the underlying vegetation. Further, prior modeling studies (Wozniak and Steiner, 2017) evaluated modeled pollen counts with observations over a different time period than analyzed here. The pollen count model can capture inter-annual changes in meteorology that drive emissions, such as temperature and wind, but cannot account for changes in plant productivity. However, one advantage to using modeled estimates is that it provides pollen concentrations based on physical processes (e.g., vegetation and environmental drivers) that are continuous in both space and time, providing more tools with which to assess risks with health data. The goal of this research, however, was not to measure the direct effects of pollen on mortality as certain variables such as medication use were not available to enable mediation analysis (see [47]). Instead our goal was to evaluate lag-temporal associations of exposure to pollens with individual health outcomes. Further research into this topic might test associations of pollen exposures on proximal outcomes such as emergency department visits for respiratory concerns, taking into account individual comorbidites and/or pharmaceutical use.

In conclusion, our study provides new evidence that specific types of pollens may be associated with increased risk for mortality from respiratory causes. With projected increases in pollen levels with climate change [48], pollen-associated health effects, including mortality, stand to increase. Efforts to prevent pollen-associated health effects should be increased, including prompt diagnosis and treatment of pollen allergies, immunotherapy, and enhanced pollen forecasting to adapt to a changing climate.

## Supplementary Information

Supplementary Material 1.

## Data Availability

Access to the data used in this research is restricted. Use of this dataset was obtained by consent of the Michigan Department of Health and Human Services (MDHHS). Both the Michigan Department of Health and Human Services. The Institutional Review Board of the University of Michigan ruled this research as exempt (HUM00166964). Those interested in obtaining these data should contact the MDHSS directly.
