# Supplementary material for: Chronic and infectious respiratory mortality and short-term exposures to four types of pollen taxa in older adults in Michigan, 2006-2017
Source: BMC Public Health. 2025 Jan 16;25:173. doi: 10.1186/s12889-025-21386-3 (PMC11737261; doi:10.1186/s12889-025-21386-3)
Supplement: Supplementary file 1 — Supplementary Material 1. [file 12889_2025_21386_MOESM1_ESM.pdf]

Supplementary Materials

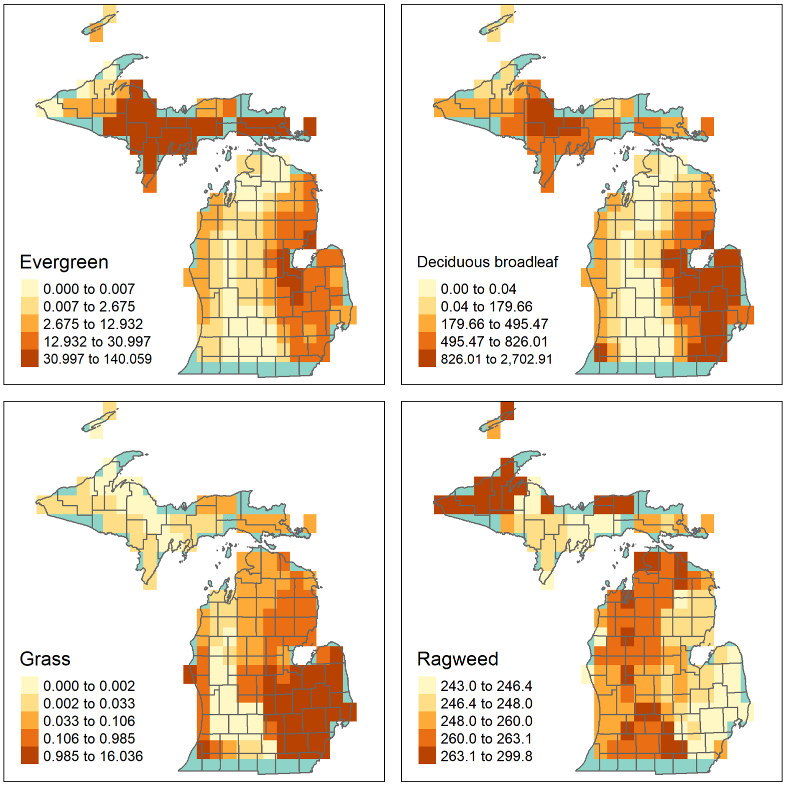

Supplementary Figure 1: Spatial distribution of the mean daily (2006-2017) modeled pollen concentrations (grains m^3; Wozniak and Steiner, 2017) throughout Michigan using the prognostic model based raster at 25km horizontal resolution, illustrating the spatial variation in pollen concentrations throughout Michigan. Internal borders represent county borders. Areas not included in the analysis are in blue. This figure is included to illustrate spatial variation in pollen exposures throughout Michigan.

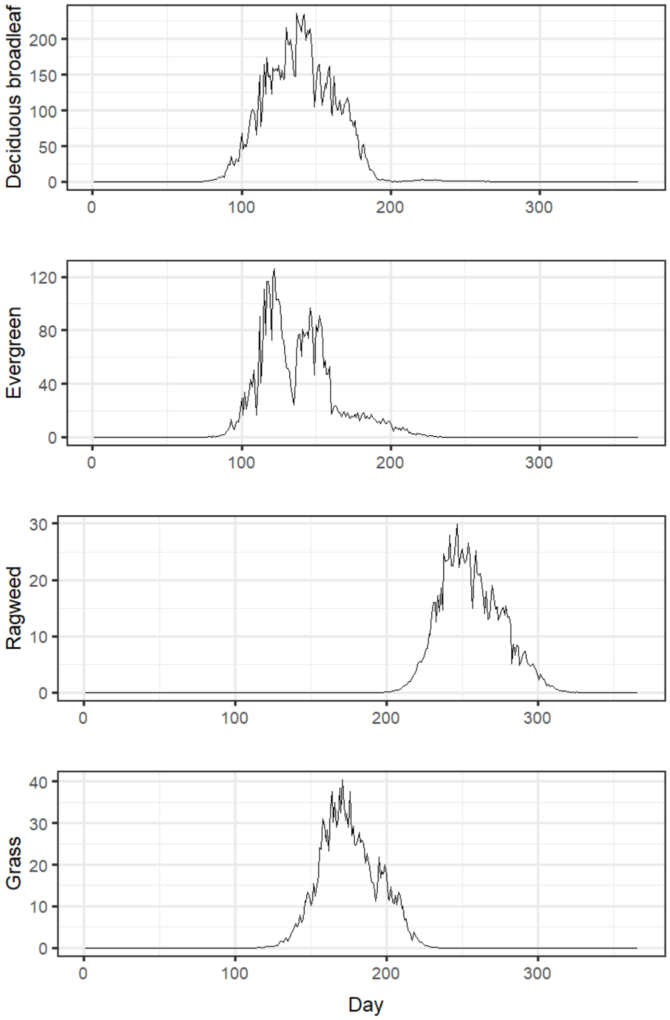

Supplementary Figure 2: Yearly patterns (daily averages from the State of Michigan for the years 2006-2017) of daily concentrations (in grains/m^3) of the four pollen taxa used in the study and average daily respiratory mortality for the entire study period.
